# Integrating Cosmic Microwave Background Readings with Celestial Navigation to Enhance Deep Space Navigation

**DOI:** 10.3390/s24113600

**Published:** 2024-06-03

**Authors:** Pedro Kukulka de Albuquerque, Willer Gomes dos Santos, Paulo Costa, Alexandre Barreto

**Affiliations:** 1 C5I Center, George Mason University, 4400 University Drive, Research Building, Fairfax, VA 22030, USA; pcosta@gmu.edu (P.C.); adebarro@gmu.edu (A.B.); 2 Aeronautics Division, Instituto Tecnologico de Aeronautica, Praça Marechal Eduardo Gomes 50, São José dos Campos 12228-900, SP, Brazil; willer@ita.br

**Keywords:** space sensor, deep space navigation, autonomous navigation system, cosmic microwave background

## Abstract

This research unveils a cutting-edge navigation system for deep space missions that utilizes cosmic microwave background (CMB) sensor readings to enhance spacecraft positioning and velocity estimation accuracy significantly. By exploiting the Doppler-shifted CMB spectrum and integrating it with optical measurements for celestial navigation, this approach employs advanced data processing through the Unscented Kalman Filter (UKF), enabling precise navigation amid the complexities of space travel. The simulation results confirm the system’s exceptional precision and resilience in deep space missions, marking a significant advancement in astronautics and paving the way for future space exploration endeavors.

## 1. Introduction

As humanity continues its quest to unravel the mysteries of the cosmos, the number of deep space missions has remarkably increased [1,2,3]. This surge in exploration is driven by an innate desire to understand our place in the Universe and seek solutions to Earth-bound challenges through the lens of space discovery. The assertion that lunar, martian, asteroid, and comet explorations could yield profitable returns for private entities has been discussed in depth in the literature [4,5,6]. Furthermore, some space agencies, like National Aeronautics and Space Administration (NASA), have outlined a clear directive to expand our reach to the moon’s surface and into the depths of space [7].

This ambitious roadmap underlines the importance of advancing deep space navigation capabilities, which remains a significant open problem in the field of astronautics [8]. The current navigation techniques for this scenario predominantly rely on Earth-based measurements to determine the spacecraft’s position [9]. However, the distances involved in deep space missions challenge the efficacy of navigational signal broadcasting. Increased distances result in more significant errors in spacecraft location determination. Additionally, greater distances reduce downlink rates, necessitating more extended periods to receive signals from spacecraft. This situation underscores the complexities and limitations inherent in long-range signal emission.

Considering these difficulties, autonomous navigation (AutoNav) has been designed to reduce dependence on systems that rely on Earth, allowing for more adaptable navigation and possibly lowering the costs and complexities of mission planning [3]. A crucial element of this approach is optical navigation (OpNav), which uses images to help the spacecraft navigate. OpNav is mainly used in two different methods [10]. First, it predicts the expected location of a target’s image in a photo based on the spacecraft and the target’s positions and velocities, camera orientation, and optical specifications. Second, it involves processing digital pictures to find the exact coordinates of the celestial bodies captured in these images.

Other innovative AutoNav methods, such as X-ray navigation (XNAV) and gamma-ray source localization-induced navigation and timing (GLINT), have been explored recently [11,12], utilizing naturally occurring signals. XNAV is a method where a spacecraft’s position and velocity are determined using X-ray signals from pulsars, calculated through the differences in time of arrival (TOA) of these signals. GLINT, focusing on gamma-ray bursts from deep space, assists in continuously updating a spacecraft’s location and movement. Unlike XNAV’s approach to calculating absolute spacecraft positioning, GLINT specializes in determining the relative distance between an observer and a reference point aligned with the direction of a distant cosmic source.

Each navigation technique presents distinct challenges and may only be suitable for some stages of a mission or different missions [13]. For instance, critical maneuvers often require real-time data, whereas extended phases like cruising emphasize utmost accuracy to minimize the need for significant corrections. The diverse requirements for precision and immediate data across various stages of deep space missions call for a navigation system that amalgamates data from multiple sources. In essence, a multi-source information fusion approach becomes indispensable [14]. Consequently, a composite navigation strategy, which harnesses sensors to assess both position and velocity, can markedly enhance the precision and reliability of deep space navigation systems. Furthermore, as of the current writing, no comparable method has been identified in the existing literature, highlighting the novelty and contribution of this work to the space field.

This paper introduces an innovative navigation system for deep space exploration that leverages the unique characteristics of the cosmic microwave background (CMB) as a novel reference signal. It is combined with celestial navigation (CeleNav) to create a comprehensive solution. This method marks a substantial advancement in navigation via signal of opportunity (NAVSOP), offering a paradigm shift towards fully autonomous navigation. By harnessing naturally occurring signals, this innovative technology equips spacecraft to ascertain their status and position independently, a capability that remains effective irrespective of the spacecraft’s distance from its starting point.

The use of the Doppler effect in reference signals for navigation is not new. Two methods stand out within the NAVSOP signals, which occur due to natural events. One utilizes the Doppler shift of the solar spectra [15], and the other employs the relativistic Doppler effect on stars [16]. Both methods face technological challenges in their implementation [16,17]. The use of the CMB differs from these methods as it employs different types of sensors, such as bolometers and radiometers, which also have technological difficulties, as is discussed later.

The structure of this article is designed to guide the reader through a comprehensive study. Section 2 delves into the theoretical underpinnings of the CMB, highlighting its pivotal role as a navigational aid in the space. Section 3 outlines translating CMB signals, considered previously filtered from foreground noise, into a quantifiable measure of spacecraft velocity. Section 4 includes a preliminary discussion on the feasibility of measuring the CMB. Section 5 introduces our innovative navigation system, which synergistically combines CMB data with traditional CeleNav techniques to enhance accuracy in deep space navigation significantly. Section 6 presents an array of simulation results alongside a discussion of the practical implications of our findings, firmly positioning our research at the forefront of astronautical science and future exploratory missions. Concluding the article, Section 7 briefly summarizes our key outcomes and outlines prospective avenues for further investigation.

## 2. The Cosmic Microwave Background as a Navigation Reference

The CMB is a pervasive thermal radiation filling every part of the observable Universe. The contemporary scientific understanding posits that the CMB we observe today originated around 380,000 years post-Big Bang, during the epoch known as recombination or decoupling [18]. Owing to the Universe’s ongoing expansion, this radiation has been red-shifted into microwave wavelengths [19]. The CMB’s unique characteristics render it a subject of profound interest in cosmology. Key among these properties is its near isotropy and consistent uniformity, akin to a blackbody’s. These characteristics have been verified with remarkable accuracy and precision by Bennett et al. [20].

Exhibiting a remarkable isotropy, the CMB presents an almost uniform radiation intensity in every direction [21]. Its isotropic nature, combined with its homogeneity—the consistency of its properties across various locations—has significant ramifications in cosmology. The CMB’s observed isotropy and homogeneity strongly support the cosmological principle, which posits that the Universe, when observed on a sufficiently large scale, appears uniform regardless of the observer’s location or direction of observation [21]. Nevertheless, achieving such a high degree of isotropy and homogeneity gives rise to a challenge known as the light–horizon problem [22].

However, it is crucial to acknowledge that the CMB is not perfectly isotropic or homogeneous. The most significant deviation results from the Doppler effect and is at a ratio of approximately 1:100,000 [23]. Other minor anisotropies have also been measured, representing fluctuations that occurred just before the Universe became transparent, capturing the attention of cosmologists [24]. These subtle anisotropies or fluctuations are paramount, as they offer insights into various cosmic attributes, including the curvature of the Universe and the presence of dark matter. The analysis of these anisotropies typically involves spherical harmonics [25]; mathematically, the CMB anisotropy expansion is represented as [18]

(1) $$\frac{\Delta T_{a}\left(\vec{x_{0}},\vec{n}\right)}{T_{0}}=\sum_{l,m}a_{lm}\left(\vec{x_{0}}\right)Y_{lm}\left(\vec{n}\right),\langle a_{\ell m}\cdot a_{\ell^{\prime }m^{\prime }}^{*}\rangle =\delta_{\ell \ell^{\prime }}\delta_{mm^{\prime }}C_{\ell },$$

where $T_{a}$ denotes the temperature anisotropies, $T_{0}$ denotes the monopole temperature, $\vec{n}$ denotes the photon direction, $\vec{x_{0}}$ is the position, $a_{lm}$ are the expansion coefficients, $Y_{lm}$ are the spherical harmonics, and $C_{l}$ is the power spectrum.

In this context, the CMB signal observed in the sky can be effectively characterized by three primary temperature components [26], as delineated by Equation (2):

(2) $$T_{CMB}=T_{0}+T_{D}+T_{F}.$$

Here, $T_{0}$ represents the isotropic temperature, $T_{D}$ accounts for the signal arising from the observer’s motion relative to the radiation field, and $T_{F}$ captures the signal attributed to the density fluctuations within the CMB shortly before the last scattering surface.

Ubiquitous blackbody radiation underlies the CMB, marked by a mean temperature characteristic of the Universe’s photon temperature today. The CMB’s temperature, approximately $T_{0}=2.7255\pm 0.0006\;K$, is remarkably uniform, showcasing almost perfect isotropy with tiny fluctuations in temperature across the sky [27]. Overlaying this is the notable CMB dipole effect, $T_{D}$, a manifestation of Doppler boosting attributable to the observer’s velocity about the CMB’s frame of reference. This dipole can be dissected into two fundamental movements: the solar system’s orbit around the Milky Way and the observer’s velocity about the solar system’s barycenter (SSB) [23]. The third crucial element consists of the CMB’s density fluctuations $T_{F}$, which, though minute, form a pattern that aligns with an isotropic and Gaussian random field, with typical variations of 100 μK [26].

In cosmology, the primary goal is to analyze the CMB’s density fluctuations, which involves data cleaning, calibrations, signal separation, and map creation for power spectrum estimation. However, for navigation purposes, the objective is another. The inquiry centers on whether determining the dipole temperature is feasible, given the knowledge of the CMB’s monopole and its fluctuations, which are approximately Gaussian and characterized by their mean and variance. A similar procedure is used for calibrating CMB sensors using the solar dipole, whose amplitude and direction are well-established from previous measurements. By measuring the dipole, a CMB instrument’s accuracy can be ensured [28]. It is important to note that the amplitude of the solar dipole, in terms of temperature fluctuation, is approximately 3.3 mK [29]. Nevertheless, before delving into the challenges and limitations of finding the dipole, it is essential to understand the relationship between the Doppler effect and the resulting anisotropy in the CMB.

The concept of an observer in a cavity immersed in a thermal bath, with velocity-dependent perceived radiation, has been discussed since the early 20th century [30]. At that time, Hasenöhrl, using Galilei transformations [31], and Von Mosengeil, using Lorentz transformations [32], computed the Doppler signal for an observer moving in a cavity. After that, Pauli [33] highlighted the problem of an observer’s motion relative to the background radiation, noting that relativity allows for determining the spectral distribution based on temperature and direction for a stationary observer. Following the measurement of CMB by Penzias and Wilson in 1965 [34], it is suggested that one could test Mach’s principle and measure the rotation of the Universe by assessing the effect of rotation on that radiation [35]. This led to the publication of several studies that derived an equation correlating the observer’s movement in a thermal bath [36,37,38,39].

Furthermore, hints of CMB’s potential as a navigation tool were discussed in the 1970s. The CMB could provide a preferred frame of reference for determining velocity [23]. According to his proposition, an observer moving relative to this preferred frame would perceive the radiation as brighter when looking in the direction of motion and dimmer in the opposite direction. An observer situated within an isotropic source of blackbody radiation at a specific temperature, moving at a certain speed relative to the preferred radiation frame and looking at a specific angle from this velocity vector, would see a blackbody spectrum at a different temperature. By fitting the measured temperature as a function of direction, one can determine the direction and magnitude of the peculiar velocity.

Therefore, by filtering the measured temperature from a moving observer in space, accounting for various forms of noise, and employing a Gaussian model to account for the fluctuations, it is possible to calculate the observer’s velocity relative to the CMB. This is achieved by comparing the known monopole and anisotropies to the measured temperature, which changes depending on the observer’s motion.

However, this task presents various challenges and limitations. Statistical instrumental noise, systematic effects due to nonidealities in measurement instruments, foreground emission from other sources, and confusion from the high-order CMB temperature fluctuations increase the uncertainty of the dipole value [26]. Another problem is the diminutive signal caused by the observer’s movement. For instance, at a velocity of 100 km/s, a temperature difference of only 2 mK occurs for a background temperature of 3 K [38].

Despite formidable technical challenges, groundbreaking space missions such as COBE, WMAP, and Planck have captured the CMB’s anisotropies thanks to their pioneering instruments and methodologies [40]. Remarkably, the precision of CMB measurements has been on an exponential incline, mirroring the pace set by Moore’s law, with sensitivities seeing a twofold increase approximately every two years since the mid-1960s [41]. This advancement underscores that the technology is still evolving and has yet to reach its peak.

The scope of this paper concentrates on applying theoretical models to enhance spacecraft navigation by estimating position and velocity rather than developing new methodologies for minimizing noise and measurement errors in CMB readings. The selection of appropriate sensors and the determination of operational frequencies, while acknowledged as critical technical factors, fall outside the main objective of this research. The idea is to go from Equation (2) to the estimation of the position and velocity of the spacecraft. Therefore, it is used as an approach for deriving velocity from the CMB signal and joining this information with others to construct a comprehensive navigation system. Furthermore, the paper compares three distinct navigation systems: one using solely CMB sensors, another based on the CeleNav method, and a hybrid system integrating both CMB and CeleNav sensors, showcasing the potential improvements in navigational accuracy these methodologies may offer.

## 3. Methodology for CMB Velocity Determination

The concept of CMB serving as a navigational signal is rooted in its unique properties and relevant theoretical frameworks. CMB’s isotropic nature, omnipresence, and inherent anisotropies resulting from the Doppler effect make it a compelling candidate for a navigational beacon for interstellar travel.

The premise, established early in the 20th century by Hasenöhrl and Von Mosengeil, that an observer moving relative to a thermal bath perceives radiation differently forms the basis of this concept [30]. However, deriving the amplitude of the effect in temperature is not straightforward. The works carried out by Condon and Harwit [36], Heer and Kohl [38], and Peebles and Wilkinson [39] achieved this derivation using transformations of energy and angles and their relationship. In this research, the derivation follows a more straightforward path, the one used in [37]. It starts evoking the Lorentz-invariance of distribution functions f(P) of particles of momentum $p\vec{n}=P$ in phase space [42].

Therefore, considering two reference systems, one fixed in the observer (*f* subscript) and the other one at rest with the last scattering surface (*s* subscript):

(3) $$f{\left(P\right)}_{f}=f{\left(P\right)}_{s}.$$

In that regard, if an observer is moving, the Lorentz transformation for the energy of the photon, ${\left(cp\right)}_{f}$, is

(4) $${\left(cp\right)}_{f}=\left(\frac{\sqrt{1-|\vec{v}{/c|}^{2}}}{1-\left(\vec{v}/c\right)\cdot \vec{n}}\right){\left(cp\right)}_{s},$$

in which *c* is the velocity of the light, $\vec{v}$ is the velocity of the observer, *p* is the magnitude of the momentum, and $\vec{n}$ is the direction of the observation.

Applying this to a blackbody distribution function and using Lorentz-invariance of distribution functions reveals

(5) $${\left(\frac{2h^{3}}{\exp\left(cp/kT\right)-1}\right)}_{s}={\left(\frac{2h^{3}}{\exp(cp/kT)-1}\right)}_{f}\to T_{f}\left(\vec{n}\right)=\left(\frac{p_{f}}{p_{s}}\right)T_{s},$$

where *h* is Planck’s constant, *k* is Boltzmann’s constant, *T* is the absolute temperature, and cp is the photon’s energy.

This implies an equation representing the directionality of the temperature measurement as

(6) $$T\left(\vec{n}\right)=\left(\frac{\sqrt{1-|\vec{v}{/c|}^{2}}}{1-\left(\vec{v}/c\right)\cdot \vec{n}}\right)T_{0}.$$

Consequently, an observer moving relative to the CMB rest frame perceives a Doppler-shifted CMB spectrum, hotter (blue-shifted) in the direction of motion and colder (red-shifted) in the opposite direction.

It is worth mentioning that in the case of a measurement made by a spacecraft on an interplanetary journey in the solar system, the velocity to be considered in Equation (6) is the sum of the spacecraft’s velocity to the SSB, ${\vec{v}}_{sp}$ and the velocity of the SSB to the last scattering surface, ${\vec{v}}_{SSB}$:

(7) $$\vec{v}={\vec{v}}_{sp}+{\vec{v}}_{SSB}.$$

The latter velocity is known to be 370 km/s toward $\left(l,b\right)=\left(264^{\circ },48^{\circ }\right)$ [43], where *l* is the galactic longitude and *b* is the galactic latitude.

Therefore, because of the nonlinearities in Equation (6), it is essential to understand the impact of variations in the variables and the effects on the temperature measurement. The two topics to highlight are velocity magnitude and velocity direction.

Increasing the velocity magnitude causes the temperature measurement to deviate further from the monopole temperature, and higher velocities imply a greater difference. A measurement more aligned with the velocity also shows a temperature deviation greater than the monopole temperature. This occurs because the velocity’s perpendicular component influences the measurement less, as inferred from Equation (6).

Expanding on Equation (2), which is a model of the CMB sky signal measured by a sensor, and incorporating Equation (6) for the term $T_{0}+T_{D}$, since it shows the deformation caused by the Doppler effect on the temperature measurement, a novel equation is formulated to establish a relationship between the spacecraft’s velocity and the temperature measurement. Including noise, η, in this model is critical, as it accounts for the inherent imperfections in foreground models that need to be extracted from the sky signal. There are other factors, including, but not limited to, systematic errors introduced during the calibration and filtering of raw sky data. Therefore, it can be noticed that

(8) $$T_{CMB}=\left(\frac{\sqrt{1-|\vec{v}{/c|}^{2}}}{1-\left(\vec{v}/c\right)\cdot \vec{n}}\right)T_{0}+T_{F}+\eta .$$

Thus, filtering techniques are crucial in data analysis for minimizing the impact of $T_{F}$ and η, significantly improving the raw data quality. This approach is instrumental in reducing noise and outliers, yielding a clearer and more accurate depiction of the underlying patterns or trends in the data. The primary aim is to distill essential information from the data while preserving its original structure. Following the application of a filter, Equation (8) can be reformulated for an estimate of the CMB’s temperature, ${\hat{T}}_{CMB}$, ready for use in estimation algorithms or for analytical solutions:

(9) $${\hat{T}}_{CMB}\approx \left(\frac{\sqrt{1-|\vec{v}{/c|}^{2}}}{1-\left(\vec{v}/c\right)\cdot \vec{n}}\right)T_{0}.$$

Addressing Equation (9) analytically poses significant challenges due to the complexity of solving nonlinear systems. This necessitates the use of advanced mathematical strategies and sophisticated computational algorithms. Techniques like optimization or iterative processes, especially those derived from the Newton method [44], are particularly effective in navigating these nonlinear equations [45]. However, the unique conditions of this analysis add layers of complexity, such as the critical $\vec{v}/c$ ratio, which could lead to a singular Jacobian matrix and significantly affect numerical accuracy, especially with values nearing zero.

Moreover, the precision of initial estimates becomes a considerable challenge, notably in the early phases of determining a spacecraft’s trajectory or position [46]. The current state of CMB measurement technology requires sensors to be directed at a specific point in space for extended periods to amplify the signal-to-noise ratio and filter out extraneous noise [20]. However, advancements in sensor technology, as described in [41], hint at the possibility of new sensing methods that could circumvent these limitations.

Given these considerations, the unscented Kalman filter (UKF) was selected for its ability to mitigate Gaussian noise while maintaining the integrity of the original dataset. Unlike other filters, the UKF does not require solving the nonlinear equations directly and offers resilience in scenarios of sporadic sensor input. This choice underscores the UKF’s versatility in handling complex data analysis tasks, offering a robust solution to the intricate problem of accurately estimating states in the presence of nonlinear dynamics and measurement uncertainties.

## 4. Preliminary Feasibility Assessment of CMB Measurements for Navigation Purposes

The potential of utilizing CMB for velocity determination is compelling; however, it faces numerous challenges and limitations. A primary obstacle is detecting the minute signal caused by the observer’s movement. Another significant challenge is distinguishing this specific anisotropy from others, as well as from various sources of noise. Microwave radiation from other origins can introduce interference, which must be carefully filtered out to ensure accurate measurements [20].

To effectively use CMB for navigation, it is essential to understand the various factors that influence its measurement. This involves developing precise models of the CMB spectrum and comprehending the impacts of cosmic expansion, local radiation sources, and emissions from the spacecraft itself [47]. While CMB presents a promising new signal for navigation, its successful application requires advanced technology and sophisticated signal detection, filtering, and interpretation techniques.

Despite these hurdles, space missions like COBE [48], WMAP [49], and Planck [50] have employed innovative instruments and scanning methods to measure anisotropies in the CMB. These missions have greatly enhanced our understanding of the Universe. However, achieving the necessary precision and accuracy for meaningful measurements remains challenging.

### 4.1. Foreground Noise

The CMB is dominant at frequencies around 100 GHz [51]. However, foregrounds in this wavelength typically constitute 10% of the CMB anisotropies [52]. Foreground contamination refers to interference from various cosmic and galactic sources that can distort or obscure the CMB signal. The primary sources of this interference include:

- Galactic Synchrotron Emission: This nonthermal radiation is produced by high-energy electrons spiraling in the Galaxy’s magnetic field [53]. It dominates at low frequencies (under 10 GHz) and can affect the CMB signal at frequencies below 50 GHz. However, its spectral index causes it to decline sharply with increasing frequency, minimizing interference above 70 GHz [51];
- Galactic Free–Free Emission: Also known as Bremsstrahlung, this radiation occurs when an electron’s path is deflected in the electric field of an ion [53]. It is predominant in the 20 to 60 GHz range and has minimal impact above 90 GHz [51];
- Spinning Dust Emission: This emission arises from the rotational motion of tiny dust grains in the interstellar medium of galaxies [53]. These spinning dust grains, typically a few nanometers in size, emit radiation mainly below 30 GHz as they interact with ambient radiation and magnetic fields [51]; and
- Galactic Thermal Dust Emission: Thermal radiation from dust grains in the Galaxy’s interstellar medium [53], which peaks around 2000 GHz but remains significant at lower frequencies [51]. Due to its thermal origin, the spectrum of this radiation resembles that of a blackbody [51].

Instruments like those that were used on missions such as COBE, WMAP, and Planck generally cannot directly distinguish between CMB and foreground photons. To address this issue, observations at multiple frequencies subtract foreground contamination and allow the isolation of the CMB signal [54]. The measurements are necessary for a wide range of frequencies, each targeting a specific type of foreground emission. By carefully analyzing and comparing signals at these different frequencies, the contributions from various foreground sources can be estimated and subtracted from the total signal through a method known as component separation [26].

In addition, the characteristics of foregrounds, such as spatial information and statistical properties that have already been modeled, can be utilized for greater sensitivity in the component separation process [55]. For example, leveraging known foreground characteristics, a machine learning process was employed to clean the data on CMB maps [56]. The Planck satellite has been pivotal in this area, observing the sky at nine different frequencies from 30 to 857 GHz, each sensitive to different foreground components [54]. The lower frequency channels effectively observed synchrotron and free–free emissions, while the higher frequencies were more sensitive to dust emission. The intermediate frequencies, particularly around 100 GHz, were most sensitive to the CMB and least influenced by foreground emissions. However, identifying and improving the accuracy of signals weaker than those obtained by Planck, by the order of tens of nanokelvin, will need additional measurements and more refined models [55].

Furthermore, polarization measurements are a powerful tool in combating noise and systematic effects [57]. Polarization refers to the orientation of oscillations in electromagnetic waves, such as light or, in this case, the CMB. Observing the polarization patterns of the CMB provides additional information that can enhance the signal-to-noise ratio, thereby improving the clarity and reliability of the signal because the CMB signal has a specific polarization pattern [58]. More importantly, polarization can help distinguish between different potential sources of a signal, such as primordial (originating from the early Universe), local (from our Galaxy or solar system), and systematic (from the instrument or observational process) [59]. Each source can imprint distinct polarization patterns on the radiation, allowing for more precise separation and extraction of the desired CMB signal.

However, measuring polarization presents challenges because it is a weaker signal than the CMB’s temperature anisotropy, necessitating more complex and refined methods [60]. Enhancing the sensitivity in the measurement of CMB polarization is a key focus for future CMB research, with new sensors being developed to achieve this goal [55].

Moreover, using the first moment, l=1, as shown in Equation (6), is not the only method to measure the observer’s peculiar velocity relative to the CMB’s last scattering surface. The observer’s movement causes modulation, altering the apparent temperature fluctuations in the direction of the velocity, and aberration, shifting the apparent arrival direction of CMB photons towards the velocity direction [61]. Consequently, measuring the peculiar velocity on the CMB using high-multipole off-diagonal correlations is possible [62]. These measurements can serve as a confirmation and correction for the velocity determined using Equation (6).

However, this method presents significant technological challenges. The Planck satellite, known for its sensitivity in measuring higher multipoles, provides an accuracy of approximately 60 km/s [61]. Achieving an accuracy on the order of 8 km/s would require a sensor capable of measuring high multipoles approximated up to l=5000, which far exceeds the capabilities of current and near-future technologies [61].

### 4.2. Systematic Errors

Understanding the nature of systematic errors in CMB experiments is crucial. These errors, arising from non-random signals in the data not caused by the sky, significantly impact the quality and accuracy of CMB data [63]. According to [64], some contributors to these systematic errors include the following:

- Gain Fluctuations: These are caused by various instrumental and environmental factors during a scan observation. They result in residual calibration uncertainty, which modulates the polarization field and can distort the CMB signal;
- Angle Errors: These arise from thermal deformation, mechanical vibration of instruments, errors in telescope-attitude determination, and inaccuracies in optical system calibration. Such errors affect the alignment of the observed data with the true sky signals; and
- Pointing Errors: These occur when there is a mismatch between the actual pointing direction and its estimated direction, degrading the spatial position accuracy of the observed data.

Addressing systematic errors implies high operational demands. Continuous monitoring and adjustment of the gain throughout the observation can minimize residual calibration uncertainties. Maintaining precise control over the thermal and mechanical stability of the instruments and accurate calibration of the optical system can reduce angle errors. Pointing errors are mitigated through sophisticated algorithms and precise attitude determination systems, ensuring the pointing direction is accurately estimated and corrected.

However, due to the limitations of the current technology, it is challenging to eliminate these errors. Increasing the number of data points is crucial to manage and minimize them [63]. A larger dataset helps average out random fluctuations, reducing the impact of systematic errors. Instead of combining data points on the spacecraft (on-board co-addition), sending all the raw data to the ground is preferable. This approach allows for more detailed and accurate data analysis on Earth, where robust computing resources are available [63]. However, this solution does not align with the autonomous use of CMB data in navigation systems, necessitating a trade-off between data points and on-board computational processing.

Complex scan patterns also play an essential role. Scans should cover each area from multiple directions to enhance error correction by averaging many systematic errors that would otherwise be difficult to separate from the signal [63]. This approach enables more uniform scanning, mitigating the impact of directional-dependent systematic effects [53]. Considering the desired sensitivity of the spin rate is important, as most radiometers have sensitivity inversely proportional to the observation time of a certain point [65].

By operationally understanding and addressing these types of systematic errors, combined with collecting a large number of data points using a scan pattern that allows for superimposed circles, researchers can improve the quality of CMB observations and enhance the accuracy of velocity determination.

### 4.3. Sensors

This subsection discusses the sensors that can measure the CMB to determine spacecraft velocity. The goal is to use simple models to obtain initial sensitivity estimates and check the technology’s feasibility.

As discussed earlier, the primary challenges related to foreground noise and systematic errors are more technological. However, further analysis and understanding of these issues, which future CMB missions can provide, are also necessary.

The information from sensors, combined with the details on foreground noise and systematic errors discussed earlier, allows us to derive some preliminary requirements for using the CMB for navigation purposes in a more realistic manner.

The first question to address is the required level of precision. To determine this, it is necessary to evaluate the sensitivity of the measurement to detect a certain variation in velocity and its effects on temperature. Using Equation (6) and transforming the variables to $\beta =|\vec{v}/c|$ and θ, the angle between the direction of observation and the velocity, the following is obtained:

(10) $$T\left(\beta ,\theta \right)=\left(\frac{\sqrt{1-\beta^{2}}}{1-\beta \cos\theta }\right)T_{0}.$$

For small variations δT and using a linear approximation only considering variations in the velocity, we obtain

(11) $$\delta T\approx \frac{dT}{d\beta }\delta \beta =T_{0}\frac{d}{d\beta }T\left(\beta ,\theta \right)\delta \beta ,$$

(12) $$\delta T\approx T_{0}\left(\frac{\cos\theta -\beta }{{\left(1-\beta \cos\theta \right)}^{2}\sqrt{1-\beta^{2}}}\right)\delta \beta .$$

Using Equation (12), it is possible to calculate the temperature variations needed, considering a requirement to detect velocity variations at least to the order of 1% of the velocity of the SSB relative to the last scattering surface. When the sensor is aligned with the velocity direction (cosθ=1), the temperature change is approximately 33.6 μ K. In contrast, when the sensor is pointed 90 degrees to the velocity direction (cosθ=0), the temperature change is significantly smaller, approximately 41.5 nK.

The second question is whether it is feasible to measure temperature variations in the microkelvin and nanokelvin range using the current sensors for measuring the CMB. To address this, two types of sensors—radiometers and bolometers—must be presented.

A standard solution for measuring the CMB with a radiometer is the Dicke radiometer equipped with a superheterodyne receiver [66]. Similar concepts have been employed in missions such as COBE [29], WMAP [20], and Planck [54].

A Dicke radiometer employs a switching mechanism to compare the signal from the antenna with a reference source, effectively mitigating gain variations and improving measurement accuracy. The superheterodyne receiver converts the high-frequency input signal to a lower intermediate frequency (IF) before amplification, reducing noise levels. The components of this system include the following:

- Antenna: Captures the CMB signal;
- Dicke Switch: Rapidly alternates between the antenna signal and a reference source, minimizing gain variations;
- Local Oscillator: Generates a stable frequency used for mixing with the input signal;
- Mixer: Combines the input signal with the local oscillator signal, producing the IF signal;
- IF Amplifier: Amplifies the lower-frequency IF signal, reducing noise compared to direct amplification; and
- Detector: Converts the amplified signal to a measurable output.

The equation used to calculate the temperature sensitivity of the Dicke radiometer is [66]

(13) $${\left(T_{A}\right)}_{rms}=\frac{2T_{sys}}{{\left(\Delta \nu \Delta t\right)}^{1/2}},$$

where $T_{sys}$ is the system noise temperature, Δν is the bandwidth, and Δt is the integration time.

Several factors affect the performance of the radiometer:

- Bandwidth: Increasing the bandwidth improves sensitivity but also increases noise. In the Planck mission, a bandwidth of approximately 20% of the central frequency, around 1 GHz for the three selected frequencies, was targeted [67]. Achieving this is feasible but requires high-quality electronics;
- System Noise Temperature: Higher frequencies lead to higher $T_{sys}$, making it impractical to use this type of equipment for frequencies above 100 GHz for CMB measurements. An appropriate $T_{sys}$ value is approximately 30 K [66]; and
- Integration Time: Longer observation times improve precision. Therefore, a data accumulator can enhance accuracy by averaging the random noise.

In summary, achieving a nanokelvin measurement with this type of sensor is mathematically feasible (Equation (13)). However, without advancements in technology that allow for increased bandwidth without a corresponding rise in noise, achieving this level of precision will require extended integration times. This approach is impractical for an autonomous navigation system, as long integration times lead to issues with storing and processing large amounts of data onboard. Additionally, the time required to achieve the desirable precision cannot be superior to the navigation time.

For microkelvin measurements, a bandwidth of 10 GHz—higher than the current technological capabilities—could achieve a short integration time of approximately 90 s, which is beneficial for the navigational method described in Section 5. With a more realistic bandwidth of 1 GHz, the required integration time increases to approximately 30 min. In this scenario, an initial data accumulation phase would be necessary, introducing complexities in dealing with the unknown velocity at the beginning and the need for an additional velocity sensor. Once sufficient data are accumulated, a similar approach to the one presented in Section 5 can be utilized. However, the data accumulation case is not explored in this study.

Therefore, we consider the other sensor, the bolometer, which is used in an extensive range of frequencies. This results from the principle of thermal detection, which is a pervasive process at nearly all wavelengths [66]. A bolometer is a device that measures the power of the incident electromagnetic radiation via the heating of a material with a temperature-dependent electrical resistance. The key components include:

- Absorber: Captures the incoming radiation and converts it into heat;
- Thermometer: Measures the temperature change in the absorber; and
- Heat Sink: Maintains a stable reference temperature.

The thermal response, δT, of a bolometer to an input radiation power, δP, can be described by the equation [68]:

(14) $$\frac{\delta T}{\delta P}=\frac{1/G_{eff}}{1+2\pi \nu \tau },$$

where $G_{eff}$ is the effective thermal conductance, τ is the time constant, and ν is the frequency. A balance must be struck between sensitivity and the time constant; it is not feasible to achieve both low sensitivity and a short time constant simultaneously. The behavior is similar to that shown in Equation (13). The primary difference with the radiometer presented lies in what limits their measurements and the optimal range for using each sensor.

Noise equivalent power (NEP) is a crucial parameter in evaluating the performance of bolometers, particularly in the context of CMB applications. NEP is defined as the amount of incident power required to produce a signal-to-noise ratio (SNR) of one in a 1 Hz output bandwidth. It quantifies the bolometer’s sensitivity to detect weak signals amid various noise sources.

In bolometers, intrinsic noise sources include photon, phonon, and Johnson noise [66]. Photon noise arises from the statistical fluctuations in the number of photons detected over a given period. For CMB applications, bolometers are typically saturated by photon noise [68]. This is because the CMB signals are incredibly faint, and the statistical fluctuations in the photon arrival rates dominate the noise landscape. Thus, minimizing photon noise is crucial for enhancing the sensitivity and accuracy of bolometric measurements in these applications. Spider-web bolometers [69] achieved, in space, sensitivities lower than the CMB photon noise level [68].

When comparing radiometers and bolometers for space measurements of the CMB, bolometers demonstrate superior performance, particularly at high frequencies (greater than 100 GHz) [68]. Bolometers are more effective in detecting faint signals from the CMB because they are not limited by the quantum noise limit like radiometers, resulting in more accurate and sensitive measurements.

However, the downside of bolometers is that they require much lower operating temperatures for the sensors, typically in the order of millikelvins, whereas radiometers only need to operate at temperatures in the order of tens of kelvins [66]. This requirement makes bolometers more challenging to implement and maintain in space missions.

The final question concerns the power consumption and mass of the instruments used to measure the CMB in the most recent space mission, the Planck mission. This question is important because it helps determine the type of spacecraft that can support a navigation system composed of these sensors. The method of average values for mass and power consumption from similar missions is taken for simplicity of analysis.

Let us reiterate the current scenario. For missions like Planck, approximately 35% of the satellite’s total power consumption and 32% of its total mass are attributed to the instruments [70]. Planck, for instance, had a total mass of 2000 kg and a power load of 1600 W [71]. This means the payload mass was estimated to be approximately 640 kg, and the power consumption was approximately 560 W.

With the current technology, a deep space navigation system using the CMB as a reference signal is only feasible for large spacecraft. However, new studies are focusing on sensors based on photonic crystal technology for CMB measurements [72]. These advancements have the potential to produce smaller and more precise instruments.

### 4.4. Preliminary Requirements

Although it is not the objective of this research to develop techniques to reduce noise and errors in reading the CMB, knowing that the choice of sensors—whether bolometers or radiometers—and frequencies is tied to more technical parameters that depend on the technology achievements, some best practices can be derived from the discussion in this section:

- Utilize multi-frequency sensors to mitigate foreground noise;
- Design sensors to be positioned on the spacecraft in a way that favors measurements aligned with the direction of its velocity;
- Implement scan patterns to mitigate systematic errors; and
- Accumulate data onboard to increase the sensitivity of the sensors.

These measures form a robust strategy for approaching CMB data collection and analysis, mitigating significant sources of interference and error. While there will always be challenges and complexities in harnessing the CMB for navigational purposes, the information gleaned from these best practices paves the way for developing a method to extract valuable data from the CMB for deep space navigation. This crucial advancement brings us closer to the day when spacecraft can navigate the cosmos using the ancient light of the Universe itself. The following section delves into this method in detail.

## 5. Integrating CMB and Celestial Navigation for State Estimation

Estimating variables is a crucial component in understanding and predicting the behavior of systems. The Kalman filter is one of the most successful tools in this arena [73]. It has been indispensable in various applications. According to [74], this is due to its unique ability to minimize the mean-squared estimation error for a linear stochastic system using noisy linear sensors under the assumption that the underlying probability distributions have finite means and second central moments.

Furthermore, the mathematical representation of the Kalman filter allows for estimating the current conditions of dynamic systems with noisy measurements and unpredictable disturbances.

Given its efficacy, it is no surprise that the Kalman filter has often been adapted to suit nonlinear problems despite its original derivation for linear systems. This adaptation has been achieved through various approximation methods, proving remarkably successful for a wide range of nonlinear problems. However, such approaches as the extended Kalman filter (EFK) are limited and can cause sub-optimal performance and sometimes divergence of the filter when there are highly nonlinear equations [75], like Equation (6).

The unscented Kalman filter (UKF) is a subset of filters developed to extend the applicability of Kalman filtering methodologies to nonlinear problems [75]. The UKF employs perturbations, much like the EKF uses perturbations for numerical approximations of partial derivatives [76]. This leads to computational requirements similar to the EKF but with improved performance for many applications with highly nonlinear behavior.

This work emphasizes the significance of the UKF for estimating spacecraft velocity, particularly in addressing the nonlinearities encountered when converting thermal radiation readings into velocity measurements. However, relying solely on velocity sensors in the measurement model for such dynamic problems may result in inaccuracies in position estimation. This is due to the cumulative velocity estimation errors over time, especially in basic state models that integrate velocity. To mitigate this issue, this research adopts a comprehensive approach that combines CMB measurements with data from additional sources, including CeleNav techniques. This integrated method aims to enhance the accuracy and stability of the spacecraft’s position and velocity estimations.

The state and measurement models, X(t) and Z(t), respectively, constitute the cornerstone of the UKF estimation process and can be represented as

(15) $$\left\{\begin{array}{c} \dot{X}\left(t\right)=f\left(X\left(t\right),t\right)+W\left(t\right)\\ Z(t)=h(X(t),t)+V(t) \end{array}\right..$$

These models in this research are developed with the spacecraft’s position (${\vec{r}}_{sp}$) and its velocity (${\vec{v}}_{sp}$) as the primary elements of the state space. Before constructing these models, it is crucial to establish the coordinate frame for spacecraft navigation as the SSB Cartesian system, where the (x,y) coordinates lie within the ecliptic plane [77]. The temporal reference point for this work is set at the epoch J2000.

Subsequently, the state dynamics of the spacecraft, which include the process noise W, are defined as follows:

(16) $$\dot{X}\left(t\right)=f\left(X\left(t\right),t\right)+W\left(t\right)\to \left[\begin{array}{c} {\dot{\vec{r}}}_{sp}\\ {\dot{\vec{v}}}_{sp} \end{array}\right]=\left[\begin{array}{c} {\vec{v}}_{sp}+w_{r}\\ {\vec{a}}_{c}+{\vec{a}}_{p}+w_{v} \end{array}\right],$$

in which ${\vec{a}}_{c}$ is the gravitational perturbations exerted by celestial bodies, and ${\vec{a}}_{p}$ is the perturbation resulting from solar radiation pressure.

The gravitational perturbations are expressed as

(17) $${\vec{a}}_{c}=\left(a_{cx},a_{cy},a_{cz}\right)=-\sum_{i}^{K}\mu_{i}\left[{\vec{r}}_{l}/{\left|{\vec{r}}_{l}\right|}^{3}\right].$$

In this context, the celestial bodies considered include the Sun and the planets within the solar system, so K=9.

The perturbation resulting from solar radiation pressure is modeled simplistically as drag and is expressed in the following manner [78]:

(18) $${\vec{a}}_{p}=\left(a_{px},a_{py},a_{pz}\right)=-C_{R}\frac{G_{1}}{{\left|{\vec{r}}_{ps}\right|}^{2}}\left(\frac{A}{m}\right){\vec{r}}_{ps}/\left|{\vec{r}}_{ps}\right|,$$

where $C_{R}$ represents the reflection coefficient, $G_{1}$ represents the solar radiation force constant at 1 AU (astronomical unit), *A* is the spacecraft’s sectional surface area, ${\vec{r}}_{ps}$ is the position vector of the spacecraft, and *m* is the spacecraft mass.

The spacecraft’s motion in this research is measured using three sensors to measure the thermal radiation of the CMB from various orientations and three optical sensors to accurately measure the angles between stars and three distinct celestial bodies. This implies two measurement groups with added noise V:

(19) $$Z\left(t\right)=h\left(X\left(t\right),t\right)+V\left(t\right)=\left[\begin{array}{c} h_{1}\left(X\left(t\right),t\right)+V_{1}\left(t\right)\\ h_{2}\left(X\left(t\right),t\right)+V_{2}\left(t\right) \end{array}\right]=\left[\begin{array}{c} Z_{1}\left(t\right)\\ Z_{2}\left(t\right) \end{array}\right].$$

The first group comprises the thermal sensors, and the measurements can be modeled as

(20) $$Z_{1}\left(t\right)=\left[\begin{array}{c} y_{1}\left(t\right)\\ y_{2}\left(t\right)\\ y_{3}\left(t\right) \end{array}\right]=\left[\begin{array}{c} \left(\frac{\sqrt{1-{\left|\left({\vec{v}}_{sp}+{\vec{v}}_{SSB}\right)/c\right|}^{2}}}{1-\left(\left({\vec{v}}_{sp}+{\vec{v}}_{SSB}\right)/c\right)\cdot {\vec{n}}_{1}}\right)T_{0}+v_{1}\\ \left(\frac{\sqrt{1-{\left|\left({\vec{v}}_{sp}+{\vec{v}}_{SSB}\right)/c\right|}^{2}}}{1-\left(\left({\vec{v}}_{sp}+{\vec{v}}_{SSB}\right)/c\right)\cdot {\vec{n}}_{2}}\right)T_{0}+v_{2}\\ \left(\frac{\sqrt{1-{\left|\left({\vec{v}}_{sp}+{\vec{v}}_{SSB}\right)/c\right|}^{2}}}{1-\left(\left({\vec{v}}_{sp}+{\vec{v}}_{SSB}\right)/c\right)\cdot {\vec{n}}_{3}}\right)T_{0}+v_{3} \end{array}\right].$$

**Figure 1:**
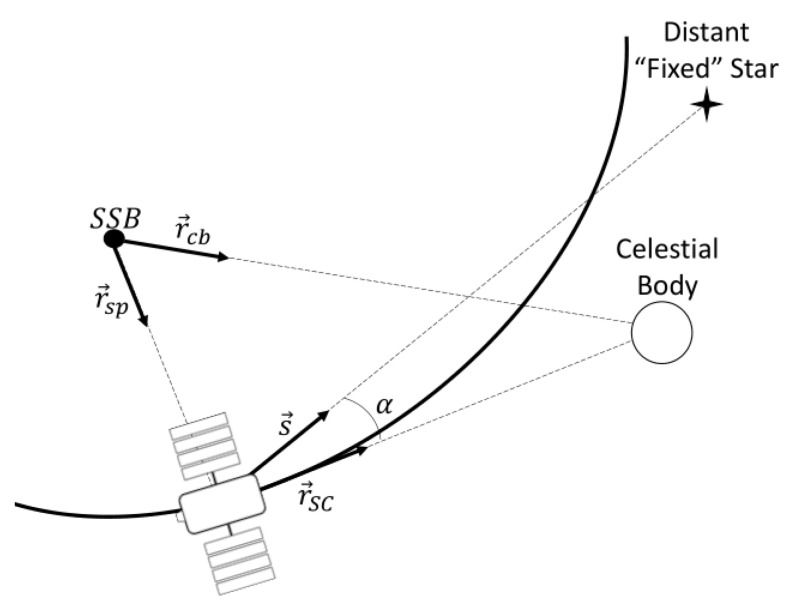
Schematic representation of Celestial Navigation (CeleNav) method.

The second group, composed of the optical sensors, uses the CeleNav method for the measurement model. It is important to note that the star’s direction vector in the SSB coordinate frame, sourced from the star catalog, closely corresponds to the direction vectors ($\vec{s}$) measured directly from the spacecraft [46]. This apparent closeness in measurements stems from the immensity distance between the star and the SSB, compared to the relatively negligible distance separating the spacecraft from the SSB. Therefore, it can be seen from Figure 1 that the angular measurement between the celestial body and the star, α, observed from the spacecraft is

(21) $$\alpha =\arccos\left(\frac{{\vec{r}}_{sc}}{\left|{\vec{r}}_{sc}\right|}\cdot \vec{s}\right)=\arccos\left(\frac{{\vec{r}}_{cb}-{\vec{r}}_{sp}}{\left|{\vec{r}}_{cb}-{\vec{r}}_{sp}\right|}\cdot \vec{s}\right),$$

where $\vec{s}$ is the direction of the star measured by the spacecraft, ${\vec{r}}_{SC}$ is the position of the celestial body relative to the spacecraft, ${\vec{r}}_{cb}$ is the position of the celestial body relative to the SSB, and ${\vec{r}}_{sp}$ is the position of the spacecraft relative to the SSB.

Using Equation (21), the second group can be modeled as

(22) $$Z_{2}\left(t\right)=\left[\begin{array}{c} y_{4}\left(t\right)\\ y_{5}\left(t\right)\\ y_{6}\left(t\right) \end{array}\right]=\left[\begin{array}{c} \arccos\left(\frac{{\vec{r}}_{cb1}-{\vec{r}}_{sp}}{\left|{\vec{r}}_{cb1}-{\vec{r}}_{sp}\right|}\cdot {\vec{s}}_{1}\right)+v_{4}\\ \arccos\left(\frac{{\vec{r}}_{cb2}-{\vec{r}}_{sp}}{\left|{\vec{r}}_{cb2}-{\vec{r}}_{sp}\right|}\cdot {\vec{s}}_{2}\right)+v_{5}\\ \arccos\left(\frac{{\vec{r}}_{cb3}-{\vec{r}}_{sp}}{\left|{\vec{r}}_{cb3}-{\vec{r}}_{sp}\right|}\cdot {\vec{s}}_{3}\right)+v_{6} \end{array}\right].$$

**Figure 2:**
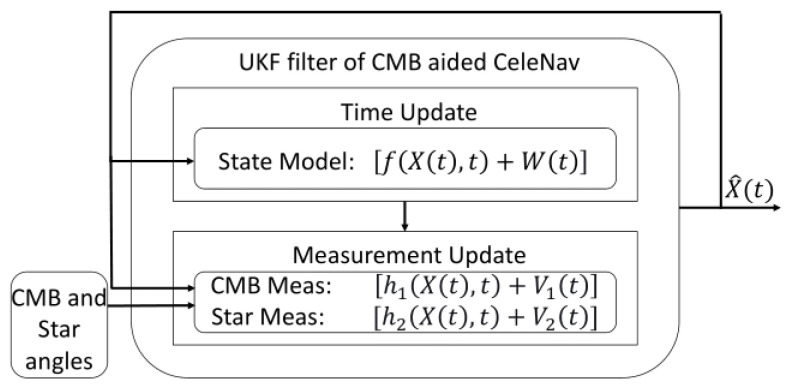
Diagram of the Unscented Kalman Filter (UKF) algorithm for Cosmic Microwave Background (CMB)-aided CeleNav.

The detailed mathematical modeling, state dynamics, and measurement equations illustrate the UKF’s robustness in handling complex navigational computations, making it an invaluable tool in aerospace engineering. This work underscores the UKF’s adaptability and precision in nonlinear estimation problems and paves the way for future advancements in space exploration and satellite navigation technologies. Integrating various sensor data and innovative techniques, as seen in Figure 2, sets a new standard in the field, promising enhanced accuracy and reliability in spacecraft trajectory prediction and positioning.

## 6. Simulation and Analysis

The intricate exploration of cosmic environments requires rigorous testing and validation procedures for the methodologies devised. In this context, simulations provide a safe and controllable environment to evaluate and fine-tune the proposed methods. The significance of the testing phase is amplified when dealing with novel approaches, such as utilizing CMB for deep space spacecraft navigation.

The primary objective of the simulations conducted in this section is to verify the feasibility and effectiveness of the proposed integrated navigation system. These simulations examine the novel concept of exploiting the CMB’s unique properties to estimate the velocity of a spacecraft in deep space.

The simulations incorporate more realistic conditions, focusing on the full scope of the navigation system. This phase considers the coordinate frame for spacecraft navigation using the SSB Cartesian coordinate system defined by (x,y,z), where (x,y) represents the plane of the ecliptic, with the center at the SSB and the reference epoch J2000. Furthermore, it entails introducing noise and a range of potential error sources into the simulation to mimic real-world environments. The aim is to evaluate the method’s capability and precision in scenarios more accurately, reflecting some real-world conditions, specifically targeting complete position and velocity estimation situations, and building upon the theoretical framework and deductions outlined in Section 5 for the UKF filter.

The results derived from these simulations elucidate the proposed method’s performance and potential, helping identify its strengths and weaknesses. This analysis also uncovers potential areas for further research and improvement, providing a solid foundation for future studies aiming to refine and optimize the proposed method.

### 6.1. Initial Conditions for the Simulation

The scenario simulated is a navigation journey from Earth to Mars. Some of the parameters and conditions of the simulation were based on similar simulations [78,79,80], which, despite having differences in sensors and applications, generally shared similar ideas. This approach was taken to validate and compare the results obtained in this study. The spacecraft’s initial position and velocity in the SSB Cartesian system are detailed in Table 1 and were determined using the Jet Propulsion Laboratory Horizons System [81]. It is important to highlight that a journey with such initial position and velocity parameters requires approximately 180 days to complete. The date considered was 15 April 2023. The spacecraft considered has a mass of 500 kg and a sectional surface area of 1 m^2^.

The reference orbit for this simulation is created using the general mission analysis tool (GMAT), considering all planetary bodies as point mass perturbations and accounting for solar flux drag. The UKF was implemented using MATLAB. An important part of this phase is the introduction of initial position and velocity errors, set at 10 km and 10 m/s, respectively, in all directions. These errors serve as the worst-case scenario. This approach examines the resilience and adaptability of the navigation system under unfavorable scenarios.

The synthetic CMB data are produced following Equation (6). The noise in this scenario is modeled as a Gaussian distribution with a zero mean and a standard deviation of 100 μK. This accounts for temperature fluctuations ($T_{F}$) and minor noise that may arise during the foreground elimination step [26].

**Figure 3:**
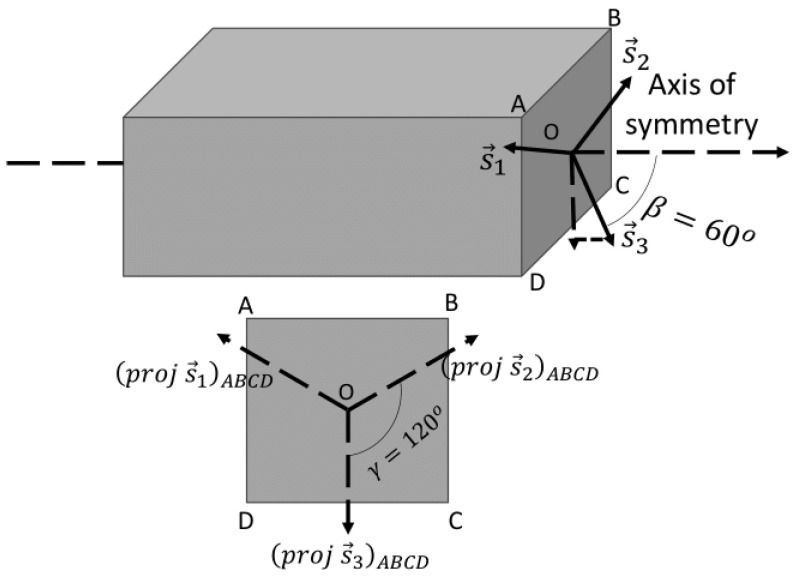
Schematic of the CMB sensor’s placement on the spacecraft.

Furthermore, three CMB sensors are considered, and the placement of these sensors is at a 60° angle relative to the spacecraft’s axis of symmetry (Figure 3). They are positioned equidistantly from each other; in this case, the angle between them is 120°.

**Figure 4:**
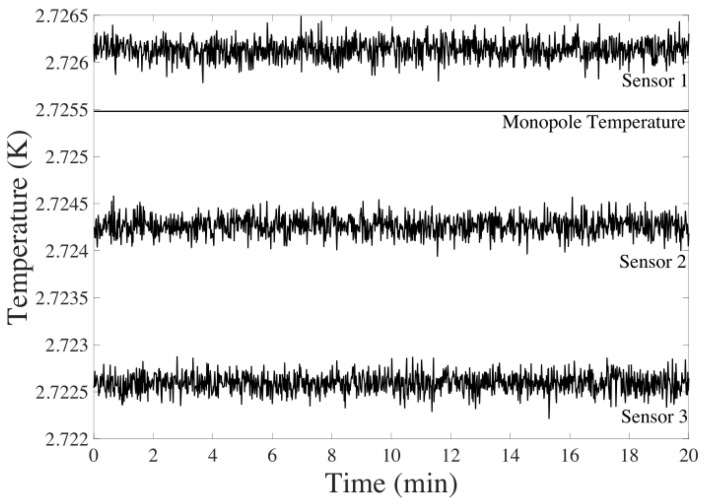
CMB signal generated with $T_{F}=N(0,$100 μk) for 20 min of flight.

An example of the data generated for three sensors can be viewed in Figure 4.

### 6.2. CeleNav Method

The first method, UKF within just CeleNav sensors, is presented to enhance the comparative analysis. This examination concentrates explicitly on utilizing Venus, Mars, and Earth as reference celestial bodies, providing a focused context for understanding the efficacy of UKF in this setting. It uses the $Z_{2}\left(t\right)$ group (as per Equation (22)) as the only measurement model.

**Figure 5:**
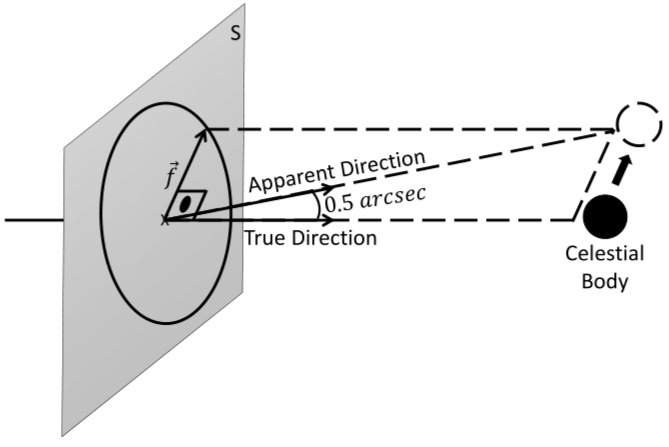
Schematics of the pointing errors applied to the CeleNav method simulation caused by mistakes in finding the center of the observed celestial body.

Notably, it introduces a pointing error to simulate the mistakes in finding the center of the observed celestial body. The error is characterized by a fixed angular magnitude of 0.5 arcseconds, with its direction, $\vec{f}$, being uniformly distributed in Plane S, as shown in Figure 5.

**Figure 6:**
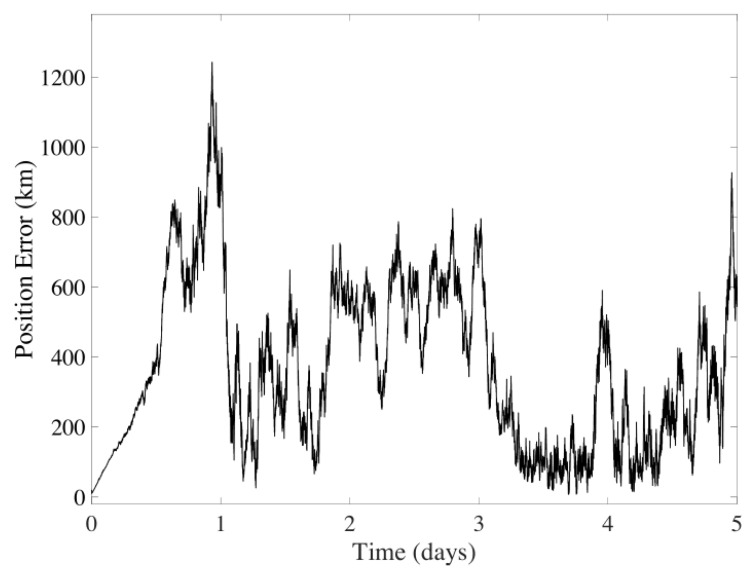
Position error in the UKF considering CeleNav sensors over five days of navigation between Earth and Mars.

**Figure 7:**
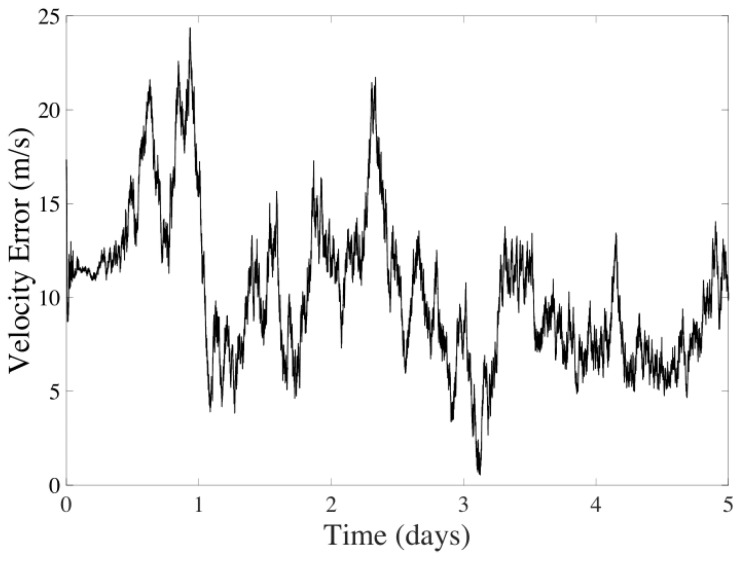
Velocity error in the UKF considering CeleNav sensors over five days of navigation between Earth and Mars.

The filter results for five days of navigation are displayed in Figure 6 and Figure 7. It is observed that the average error for the position is 345.0031 km, and for velocity, it is 9.1389 m/s, discarding the instabilities of the filter during the first day. The method’s performance shows significant variability due to errors and the spatial gap between the spacecraft and celestial bodies.

Accuracy enhancement is possible by using closer, smaller celestial bodies like Mars’ moons as reference points, which can significantly minimize errors in pinpointing the centers of celestial bodies and in distance measurement, as highlighted in [78]. However, their applicability is restricted chiefly to Mars-focused missions. This research maintains a broader, more generalized approach to ensure a more universally applicable navigation method, aiming to develop a versatile and efficient system across the solar system, thereby maximizing its usefulness in various space exploration contexts. Therefore, it will not be made a particular enhancement to the actual method.

### 6.3. CMB Method

Next, the UKF filter using only CMB sensors is presented. Consequently, the $Z_{1}\left(t\right)$ measurement model is exclusively employed (Equation (20)). The synthetic data used for these sensors are consistent with what is depicted in Figure 4.

**Figure 8:**
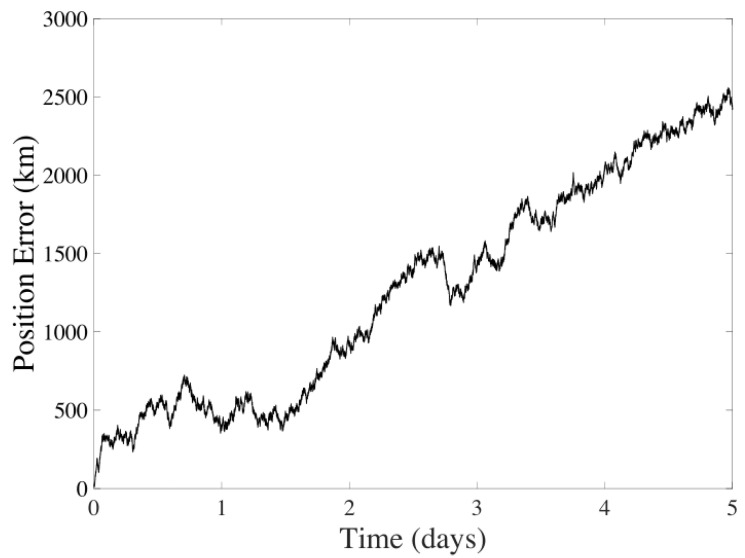
Position error in the UKF considering CMB sensors over five days of navigation between Earth and Mars.

**Figure 9:**
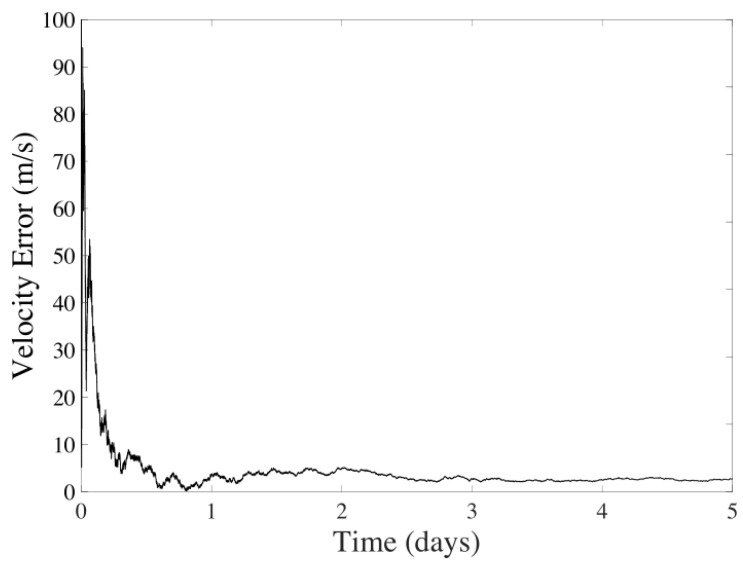
Velocity error in the UKF considering CMB sensors over five days of navigation between Earth and Mars.

As shown in Figure 8 and Figure 9, there is a noticeable decrease in velocity error over five days while the positional data diverges. It is observed that the average error for the position is $1.4664\times 10^{3}$ km, and for velocity, it is 3.0376 m/s.

The divergence is attributed to the method relying solely on measurements related to velocity and the simplistic spacecraft’s state dynamics model. In this model, position is determined through a straightforward integration of velocity, which leads to an accumulation of errors over time. Incorporating position-related measurements into the model could correct this error, but such data are unavailable in the current setup.

An important observation is the sensors’ ability to provide accurate velocity data. In this context, there is no need for a filter to smooth the data, as the current noise level is within the filter’s handling capacity. However, in scenarios where noise types significantly deviate from the Gaussian assumption for $T_{F}$ or η in Equation (8), adopting a dual estimation process, as explored in [75], can become the solution. This work will not explore this, but it can be a starting point for further studies.

### 6.4. Hybrid Method

**Figure 10:**
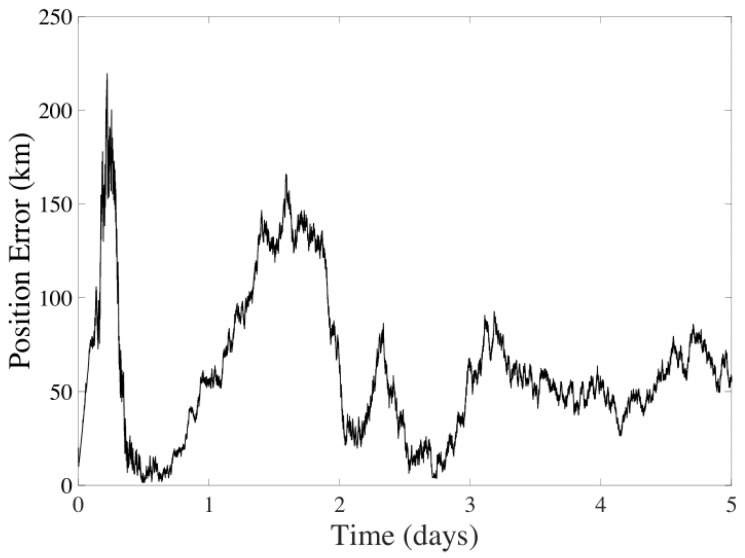
Position error in the UKF considering CMB-aided CeleNav over five days of navigation between Earth and Mars.

**Figure 11:**
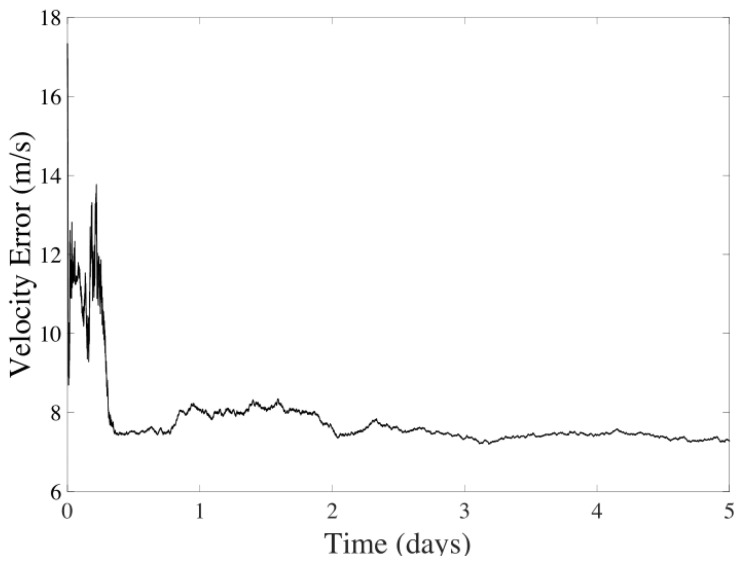
Velocity error in the UKF considering CMB-aided CeleNav over five days of navigation between Earth and Mars.

The concluding navigation strategy combines CMB and CeleNav sensors, as depicted in Figure 2. As shown in Figure 10 and Figure 11, the position and velocity filtering outcomes highlight the UKF’s adeptness at deducing state variables from complex models and sensor inputs. While variations are more pronounced in the position data, a decreasing trend in instability is observed for both variables.

This trend underscores the filter’s effectiveness in dynamically estimating states, even within nonlinear systems. Notably, the position error trends demonstrate a move towards stabilization in the initial fluctuations, showcasing the UKF’s capability to handle transient errors and maintain consistent error rates. This stabilization is significantly attributed to the accurate velocity estimations provided by the CMB sensors, which aid the CeleNav sensors in determining a more stable position value. Such evidence solidifies the UKF’s precision and dependability in state estimation amid the challenges posed by nonlinear dynamics and measurement noise complexities.

### 6.5. Analysis of the Three Methods

Before concluding, verifying whether the simulation results are consistent with other simulations is important. In this research, the position and velocity errors were found to be consistent with those reported in other studies. For example, celestial navigation using stellar spectra shift velocity measurements [80], X-ray-pulsar-aided CeleNav [78], differential X-ray-pulsar-aided CeleNav [78], Doppler-measurement-aided CeleNav [79], and differential Doppler-measurement-aided CeleNav [79] demonstrated similar results. Although the specific values vary based on the characteristics of each navigation system and certain specifics of this research, these comparisons generally support the concordance of the results, helping to verify that no gross simulation errors occurred.

Moreover, it is pertinent to juxtapose the three navigation strategies examined herein. The trio comprises the UKF with CeleNav sensors alone, the UKF with only CMB sensors, and the UKF integrating both CMB and CeleNav sensors. The evaluation is primarily based on the mean error and standard deviation concerning position and velocity, as shown in Table 2. Moreover, an assessment of the inherent pros and cons of each strategy is in order.

The first approach, leveraging CeleNav sensors, employs the $Z_{2}\left(t\right)$ measurement model (Equation (22)) to capture the orientation and positions of celestial bodies, thus providing detailed data for state estimation. This technique results in mean position and velocity errors of 345.0031 km and 9.1389 m/s, alongside standard deviations of 208.3662 km and 3.1666 m/s, respectively. Although this strategy enhances position accuracy over the second method, it can encounter difficulties in accurately determining the centers of celestial bodies, as highlighted in [78]. Moreover, it necessitates selecting orbits that offer clear visibility of celestial bodies, potentially restricting orbital options.

The second approach utilizes only CMB sensors and follows the $Z_{1}\left(t\right)$ measurement model (Equation (20)), capitalizing on the isotropic characteristics of CMB radiation. This facilitates sensor orientation that is aligned with mission goals without limiting orbit selection. It achieves notable accuracy in velocity estimation, with a mean error of 3.0376 m/s and a standard deviation of 0.7970 m/s. However, its disadvantage lies in the position estimation, leading to a significant accumulated error that extrapolates filter capacity, as indicated by a divergence behavior.

The integration of CMB and CeleNav sensors in the hybrid approach leverages the Z(t) measurement model (Equation (19)), effectively combining the strengths of both types of sensors to mitigate their weaknesses. This collaborative strategy significantly enhances the stability of state variable estimations. Notably, it results in a five-fold decrease in the mean and standard deviation of position error compared to using CeleNav sensors alone. Compared with using CMB sensors exclusively, the hybrid approach demonstrates superior performance, as the latter scenario leads to filter divergence and suboptimal results. The fusion of data from both sensor types consequently yields state estimations for positions prominent to those obtained independently by either sensor type.

For velocity estimation, the hybrid method shows a remarkable improvement in the error standard deviation, almost eleven times better than the CeleNav-only approach and three times better than the CMB-only method. However, the mean velocity error presents a mixed picture: it improves from 9.1389 m/s to 7.6495 m/s compared to the CeleNav-only method. Still, it shows a decline in accuracy from 3.0376 m/s to 7.6495 m/s compared to the CMB-only approach, which is attributed to the higher error rates of CeleNav sensors. Despite this, the hybrid method yields a more stable outcome for velocity estimation. This approach not only boosts the overall accuracy of navigation but also validates the practical application of CMB data in real-time navigation, showcasing the potential of a synergistic sensor strategy.

The hybrid UKF approach also promotes operational adaptability. While the CeleNav sensors might falter during specific mission phases, such as when obstructed by planetary shadows or poorly aligned celestial bodies, the CMB sensors maintain consistent performance. Thus, the integration ensures seamless state estimation for the entire mission duration.

Summarizing these findings, the CeleNav method shows a reasonable performance but necessitates orbits with conditions that ensure the visibility of enough celestial objects for navigation; unsuitable orbits may hinder this. The CMB-focused UKF strategy excels in velocity estimation, albeit with limitations in position estimation. Conversely, the integrated UKF method offers a balanced and effective solution, enhancing data utility, reducing errors in position estimation, and ensuring more consistent velocity measurements. This approach also provides a fail-safe mechanism by compensating for potential sensor failures. Its resilience and versatility make it a compelling choice for mission planners seeking the highest navigational accuracy and reliability levels.

## 7. Conclusions

In essence, this study addresses the complexities of deep space navigation by utilizing CMB signals. It introduces a pioneering navigation system that enables spacecraft to autonomously determine their status, independent of their position within the solar system. Although not extensively tested in various solar system locales, the theoretical underpinnings suggest its universal applicability. The work demonstrates the system’s feasibility and efficiency by exploiting the unique characteristics of the CMB as a novel navigational beacon. This is further enhanced by integrating CMB data with CeleNav techniques, showcasing a harmonious blend of traditional and avant-garde methods for space exploration.

Looking ahead, the scope for further advancement is vast. Prospective developments could explore advanced technologies and operational systems suitable for next-generation CMB navigation frameworks. Enhancing the system’s robustness in high-noise environments through a dual estimation filter is a promising avenue. Additionally, investigating alternative strategies for sensor data integration could significantly refine the navigation system’s precision and dependability.

## Figures and Tables

**Table 1:** The initial position and velocity for both simulation phases considering the coordinate frame for spacecraft navigation as the SSB ecliptic J2000 system.

State Variable	Value
Position [km]	[−1.3765 × 10^8^, −6.2494 × 10^7^, 3.2994 × 10^3^]
Velocity [km/s]	[−15.2727, −26.2104, −0.3666]

**Table 2:** The mean and standard deviation for the error in the state variables for different navigation approaches from day 1 to 5.

Sensors	State Variable	Mean Error	Std Error
CMB + CeleNav	Position	66.7610 km	38.4788 km
CMB + CeleNav	Velocity	7.6495 m/s	0.2892 m/s
CMB	Position	$1.4664\times 10^{3}$ km	639.1644 km
CMB	Velocity	3.0376 m/s	0.7970 m/s
CeleNav	Position	345.0031 km	208.3662 km
CeleNav	Velocity	9.1389 m/s	3.1666 m/s

## Data Availability

The original contributions presented in the study are included in the article; further inquiries can be directed to the corresponding author.
